# Paternal Body Mass Index (BMI) Is Associated with Offspring Intrauterine Growth in a Gender Dependent Manner

**DOI:** 10.1371/journal.pone.0036329

**Published:** 2012-05-03

**Authors:** You-Peng Chen, Xiao-Min Xiao, Jian Li, Christoph Reichetzeder, Zi-Neng Wang, Berthold Hocher

**Affiliations:** 1 Department of Infectious Diseases, the first Affiliated Hospital of Jinan University, Guangzhou, China; 2 Department of Obstetrics and Gynecology, the first Affiliated Hospital of Jinan University, Guangzhou, China; 3 Institute of Nutritional Science, University of Potsdam, Nuthetal-Potsdam, Germany; 4 Center for Cardiovascular Research/Institute of Pharmacology, Berlin, Germany; Brigham and Women's Hospital and Harvard Medical School, United States of America

## Abstract

**Background:**

Environmental alternations leading to fetal programming of cardiovascular diseases in later life have been attributed to maternal factors. However, animal studies showed that paternal obesity may program cardio-metabolic diseases in the offspring. In the current study we tested the hypothesis that paternal BMI may be associated with fetal growth.

**Methods and Results:**

We analyzed the relationship between paternal body mass index (BMI) and birth weight, ultrasound parameters describing the newborn's body shape as well as parameters describing the newborns endocrine system such as cortisol, aldosterone, renin activity and fetal glycated serum protein in a birth cohort of 899 father/mother/child triplets. Since fetal programming is an offspring sex specific process, male and female offspring were analyzed separately. Multivariable regression analyses considering maternal BMI, paternal and maternal age, hypertension during pregnancy, maternal total glycated serum protein, parity and either gestational age (for birth weight) or time of ultrasound investigation (for ultrasound parameters) as confounding showed that paternal BMI is associated with growth of the male but not female offspring. Paternal BMI correlated with birth parameters of male offspring only: birth weight; biparietal diameter, head circumference; abdominal diameter, abdominal circumference; and pectoral diameter. Cortisol was likewise significantly correlated with paternal BMI in male newborns only.

**Conclusions:**

Paternal BMI affects growth of the male but not female offspring. Paternal BMI may thus represent a risk factor for cardiovascular diseases of male offspring in later life. It remains to be demonstrated whether this is linked to an offspring sex specific paternal programming of cortisol secretion.

## Introduction

Low birth weight is an independent risk factor for cardiovascular disease and glucose intolerance in later life. This was first elucidated in a study investigating thef the geographical distribution of heart disease in Great Britain [1]. Several epidemiological studies in Europe, the United States and India have thereafter confirmed these findings [2]–[8]. It was suggested that coronary artery disease (CAD), glucose intolerance and hypertension arise from mechanisms primarily related to factors originated in the mother. In particular maternal malnutrition occurring during critical time windows of development can lead to irreversible morphological (for example: structural changes of the hypothalamic nuclei, changes in size and number of islets in the endocrine pancreas or changes of the nephron number in the kidney) and functional changes (changes of blood pressure regulating endocrine systems like the renin-angiotensin-aldosteron system) and a higher susceptibility to disease later in life (such as: hypertension, chronic renal failure, metabolic syndrome, type 2 diabetes, stroke and myocardial infarction) [9], [10]. In the context of our study, it is important to note that the low birth weight phenotype is associated with higher central (abdominal) adipositas in later life.

Meanwhile other maternal mechanisms leading to an increased risk of diabetes, renal diseases and cardiovascular diseases in later life have been described such as maternal genes modifying offspring physiology in early life without being transmitted to the offspring [11], [12] and factors affecting placenta function such as placental 11 beta HSD activity [13], [14]. Besides it is known that maternal over nutrition/obesity induces fetal programming. For example, a high protein diet offered to rat dams during pregnancy and lactation causes gender specific effects in the offspring. Male offspring is characterized by an elevated blood pressure, whereas female offspring gains more weight and has an increased energy expenditure [15]. Maternal high-fat or cholesterol over-feeding during pregnancy and lactation in rodents results in an offspring phenotype that closely resembles the human metabolic syndrome [16]. Phenotypic characteristics of the offspring in these rodent models of fetal programming included an abnormal glucose homeostasis [17], [18], increased blood pressure, abnormal serum lipid profiles and increased adiposity [19].

However, in very recent animal studies evidence emerged that paternal factors may influence fetal growth (assessed by measuring birth weight) and the risk of cardiovascular diseases in later life. Especially paternal obesity may cause long-term effects on the metabolism of the offspring. A recent study demonstrated that a chronic high-fat diet in male rats programs β-cell dysfunction in female rat offspring [20]. This study is in line with a study in humans showing that paternal body fat is a long-term predictor of changes in premenarch body fat of girls [21]. In this context it is of interest that a recent animal study also reported paternal transgenerational genetic effects besides the well-known epigenetic effects on body weight and food intake [22]. In line with this study is a human epidemiological study showing that paternal BMI (and maternal BMI) is positively correlated with childhood BMI at age of 11 and in adulthood [23]. However, little is known about fetal growth during intrauterine life in relationship to paternal obesity in humans. We thus analyzed the relationship between measures of paternal BMI, as a surrogate of obesity, and birth weight as well as ultrasound parameters describing the newborn's body shape in a Chinese birth cohort to test the hypothesis that paternal BMI may be associated with fetal growth.

## Methods

### Clinic data collection

Participants from this study came from the Guangzhou birth cohort [24]. The study was approved by the ethics committee of Jinan University, Guangzhou, China. We obtained written informed consent from all participants involved in our study.

**Table 1:** Detailed descriptive data of the newborn's mothers and fathers (n = 899).

**Maternal age, years**	28.49±3.71
**Hypertension before pregnancy, n (%)**	5 (0.56%)
**Hypertension during pregnancy, n (%)**	39 (4.33%)
**Gestational age at delivery, days**	274.44±9.79
**Gravidity, n**	1.74±1.06
**Parity, n**	1.19±0.45
**Maternal height, cm**	159.9±4.7
**Maternal BMI before pregnancy, kg/m^2^**	20.2±2.4
**Maternal weight before pregnancy, kg**	51.7±6.9
**Maternal weight at delivery, kg**	67.6±8.3
**Maternal cortisol, nmol/L**	806.87±333.34
**Maternal aldosterone, ng/ml**	0.289±0.248
**Maternal rennin activity, ng/ml/h**	5.87±3.97
**Maternal glycated serum protein, µmol/L**	117.88±29.27
**Maternal fasting glucose, mmol/L**	4.76±0.63
**Paternal age, years**	31.36±4.62
**Paternal height, cm**	171.54±5.25
**Paternal weight, kg**	68.59±9.87
**Paternal BMI, kg/m^2^**	23.28±2.88

**Data are given as mean ± SD or absolute numbers.**

We invited a total of 932 Chinese women who delivered their babies at the obstetric department of the first affiliated hospital of Jinan university between March 2010 to October 2010 to participate in the study. Inclusion criteria were as follows: (1) the newborn was born without congenital anomalies; (2) singleton pregnancy; (3) no hepatitis B, C and D, no HIV and no syphilis. After exclusion of cases that did not fulfill the inclusion criteria or were not willing to participate, we finally included 889 remaining cases. After obtaining written consent, a structured medical history was taken. Chinese guidelines for medical follow-up in pregnancy comprise a perinatal health manual which contains essential data about the pregnancy. The data in the ‘Perinatal health manual’ were also used to judge whether the women fulfilled all inclusion and exclusion criteria. The following data were extracted into our database: nationality/ethnic background, age, body height, body weight before and during pregnancy, gravidity, parity, gestational age at delivery, smoking before/during pregnancy, and alcohol consumption during pregnancy and blood pressure readings at all follow-up visits. The criteria for diagnosis of pregnancy-induced hypertension were based on the definition of the Chinese hypertension society: systolic blood pressure of ≥140 mm Hg and/or a diastolic blood pressure of ≥90 mm Hg after 20 weeks gestation, measured at least at two occasions with a break of 4 to 6 hours in-between. Gestational age was calculated from the first day of the last normal menstrual period and confirmed by either first or early second trimester ultrasound scans. Biometric data of the newborns were routinely measured immediately after delivery.

### Sample collection and glycated serum protein (GSP) assay

Fetal blood was collected from the umbilical cord (umbilical vein) within 10 minutes after delivery. Midwives collected maternal blood from a cubital vein in the delivery room or on the ward before delivery immediately after the pregnant women were hospitalized for giving birth. Mothers were asked to stop eating when being hospitalized for giving birth. Food intake prior to hospitalization was not recorded. Samples were processed immediately. The resulting plasma samples were stored at −20°C until analysis.

Total cortisol was measured by Fluorescence Polarization Immunoassay (FPIA) (Abbott, USA, Catalog number 2G98 217-232 6/05) on the AxSYM System (Abbott, USA), as performed previously [25]. Glycated serum protein was measured as recently described [24]. Plasma renin activity (PRA) and plasma aldosterone were measured by radioimmunoassay (RIA; Beijing north institute of biological technology, Beijing, China, CAT number: D01PZB for PRA measurements (angiotensin I RIA, D03PZB for aldosterone measurements), on a GC-1200γ radioimmunoassay counter (Anhui ustc zonkia scientific instruments co., LTD, China). The PRA method is based on the analysis of the generation of angiotensin I. For detail of the PRA method and aldosterone, see: Chen YP, Li Y, Wang NZ, Reichetzeder, Xu H, Gong J, Chen GJ, Pfab T, Xiao XM, Hocher B. Angiotensin Aldosterone System and Glycemia in Pregnancy. Clin Laborartory 58(May–June); (2012), in press. All the blood samples were measured by experienced technologists in a certified laboratory of the hospital.

### Ultrasound measurements during pregnancy

Ultrasound measurements during pregnancy were done as described previously [24]. All the anthropometric parameters of the newborns were measured in two types of three-dimensional ultrasound examination units with an integrated image analysis software (MEDISON ACCUVIX V20, South Korea and GE VOLUSON 730 EXPERT, USA). Only obstetric physicians that passed the examination for ultrasound diagnostics during pregnancy performed the analysis ensuring the quality of the data. The biparietal diameter was measured from the outer edge of the parietal bone near the probe to the inner edge of the other side of the parietal bone at the cross-sectional plane of the fetal brain with major land marks including the cavum septi pellucidi, thalamus, third ventricle and ambient cistern. The head circumference was measured in the same plane as the biparietal diameter using the elliptic function of the ultrasound instrument. Abdominal circumference was measured in a plane at the level of the fetal umbilical plexus perpendicular to the spine including the spine, stomach bubble, liver, umbilical vein, the skin and subcutaneous fat. Femur length was measured using predefined anatomic points that can easily be detected by ultrasound techniques in a standartizised manner (from the greater trochanter to the lateral condyle). Assessment of pectoral diameter and abdominal diameter were also conducted using standard clinical measurement protocols.

### Data analysis

Data were analyzed with SPSS version 19.0. Results are presented as mean ± standard deviation. When examining the correlation between paternal BMI with birth weight or ultrasound parameters describing fetal growth, factors that are known to influence intrauterine growth and showed a significant bivariate correlation or association with birth weight or ultrasound parameters in our cohort, respectively, were used as co-variables. Multivariable regression analysis, see also references 24 and 25. was used to adjust for confounding variables known to independently influence birth weight and intrauterine growth.

To ensure that the findings are robust, we tested the effect of paternal BMI on fetal growth in different two-sided multivariable regression models. Model A considered – besides maternal BMI – maternal and paternal age, hypertension during pregnancy, maternal glycated serum protein, parity and either gestational age (for birth weight) or time of ultrasound investigation (for ultrasound parameters) as confounding factors. Model B was calculated excluding cases of paternal hypertension but considering maternal BMI, paternal and maternal age, hypertension during pregnancy, maternal glycated serum protein, parity and either gestational age (for birth weight) or time of ultrasound investigation (for ultrasound parameters) as confounding factors. Model C considered maternal BMI, hypertension during pregnancy, maternal glycated serum protein and either gestational age (for birth weight) or time of ultrasound investigation (for ultrasound parameters) as confounding factors. A p-value of less than 0.05 was considered significant.

## Results

### Description of the cohort

Demographic data describing the participating couples and their offspring are given in table 1 and 2. We finally included 899 father/mother/child triplets with 492 newborn boys and 407 newborn girls, respectively. Participating fathers were slightly older and had a higher body mass index as their female partners. The mean age of the fathers was 31.36 years and their mean body mass index was 23,28 kg/m^2^, whereas the mean age of the mothers was 28.49 years and the mean BMI prior to pregnancy was 20.20 kg/m^2^.

**Table 2:** Biometric data of the newborns (n = 899) data of the newborns (n = 899).

**Child birth weight, g**	3260.5±441.0
**Gestational age at delivery, day**	274.4±9.8
**Child sex, male/female**	492/407
**Apgar score at 5 min**	9.94±0.28
**Apgar score at 10 min**	9.98±0.14
**Late ultrasound analysis time, day**	266.8±15.1
**Late biparietal diameter, mm**	93.8±5.0
**Late head circumference, mm**	329.1±14.5
**Late pectoral diameter, mm**	90.1±7.4
**Late abdominal diameter, mm**	102.4±8.9
**Late abdominal circumference, mm**	336.2±24.4
**Late femur length, mm**	70.4±4.2
**Fetal cortisol, nmol/L**	295.18±210.71
**Fetal aldosterone, ng/ml**	0.383±0.225
**Fetal renin activity, ng/ml/h**	7.026±4.000
**Fetal glycated serum protein, µmol/L**	90.84±20.515

Data are given as mean ± SD or absolute numbers.

### Effects of paternal BMI on fetal growth

Since fetal programming is an offspring gender dependent process [15], the analyses of the effect of maternal and paternal BMI on fetal growth were performed separately for girls and boys.

Gestational age at delivery was 274.44±9.79 days; the ultrasound examination was done at gestational day 266.8±15.1. We used these data to describe the newborns body shape, since the birth process is known to affect certain anthropometric parameters (e.g.: head circumference) immediately after birth [26].

When analyzing cases including male offpspring only, all tested models revealed that paternal BMI correlated significantly with birth weight, biparietal diameter, head circumference, abdominal diameter, abdominal circumference and pectoral diameter (Table 3).

**Table 3:** Multivariable regression analysis of the association between ultrasound measurements in late gestation or birth weight and paternal BMI in newborn boys.

		Model A	Model B	Model C
**Birth weight**	T	2.495	2.419	2.775
	B	19.535	19.690	21.381
	P-Value	0.013	0.016	0.006
	95%CI	4.126–34.945	3.661–35.719	6.215–36.547
**Biparietal diameter**	T	3.393	2.775	5.492
	B	0.247	0.246	0.272
	P-Value	0.001	0.002	<0.001
	95%CI	0.104–0.391	0.095–0.397	0.130–0.414
**Head circumference**	T	2.779	3.101	3.014
	B	0.587	0.696	0.627
	P-Value	0.006	0.002	0.003
	95%CI	0.171–1.002	0.254–1.139	0.217–1.036
**Abdominal diameter**	T	2.217	2.343	2.237
	B	0.341	0.368	0.340
	P-Value	0.027	0.020	0.026
	95%CI	0.038–0.644	0.059–0.677	0.041–0.639
**Abdominal circumference**	T	3.049	3.288	3.226
	B	1.019	1.133	1.065
	P-Value	0.003	0.001	0.001
	95%CI	0.361–1.677	0.454–1.812	0.415–1.715
**Pectoral diameter**	T	2.036	2.203	2.096
	B	0.297	0.338	0.302
	P-Value	0.043	0.028	0.037
	95%CI	0.010–0.585	0.036–0.640	0.018–0.586
**Femur length**	T	0.957	0.984	1.114
	B	0.055	0.060	0.064
	P-Value	0.339	0.326	0.266
	95%CI	−0.058–0.169	−0.060–0.180	−0.049–0.176

B: partial regression coefficient; 95% CI: 95% confidence interval for B; T: standardized regression coefficient.

Different regression models showing the association between ultrasound measurements in late gestation or birth weight and paternal BMI in newborn boys. Shown are the T, B and P-values for paternal BMI. B: non-standardized regression coefficient.

Model A: multivariable regression analyses was done in all cases considering maternal BMI, paternal and maternal age, hypertension during pregnancy, maternal glycated serum protein, parity and either gestational age (for birth weight) or time of ultrasound investigation (for ultrasound parameters) as confounding factors.

Model B: multivariable regression analyses was done excluding cases where fathers had hypertension but considering maternal BMI, paternal and maternal age, hypertension during pregnancy, maternal glycated serum protein, parity and either gestational age (for birth weight) or time of ultrasound investigation (for ultrasound parameters) as confounding factors.

Model C: multivariable regression analyses was done in all cases considering maternal BMI, hypertension during pregnancy, maternal glycated serum protein, and either gestational age (for birth weight) or time of ultrasound investigation (for ultrasound parameters) as confounding factors.

**Figure 1:**
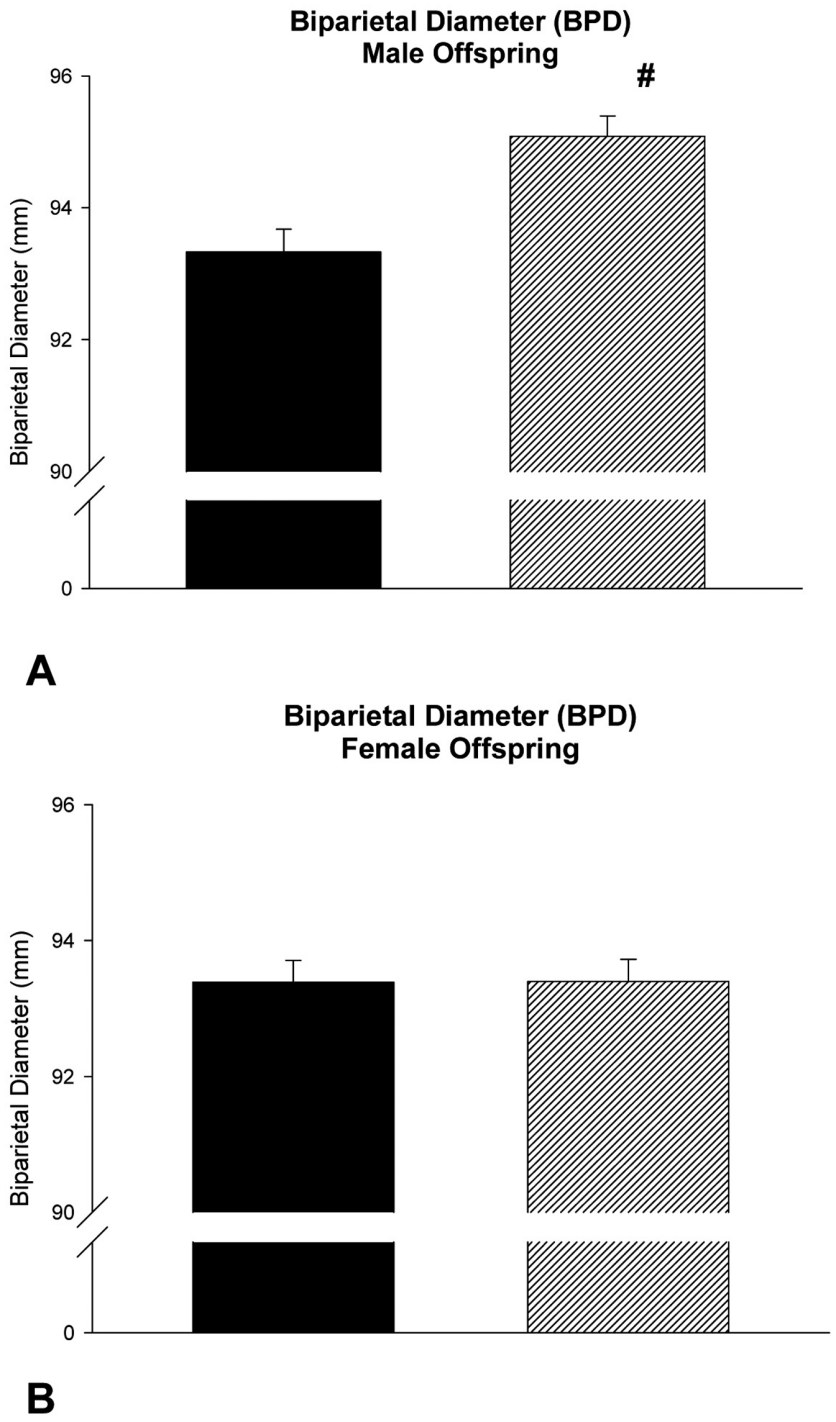
Biparietal diameter of male (A) and female (B) offspring in relationship to paternal BMI. Ultrasound examination was done in average at gestational day 266.8. Black Bar: Paternal BMI below median; Hatched Bar: Paternal BMI above median. #: p = 0.008 versus male offspring of fathers with a BMI below the median of all paternal BMI data of the cohort; Data are given as mean +/− SEM.

**Figure 2:**
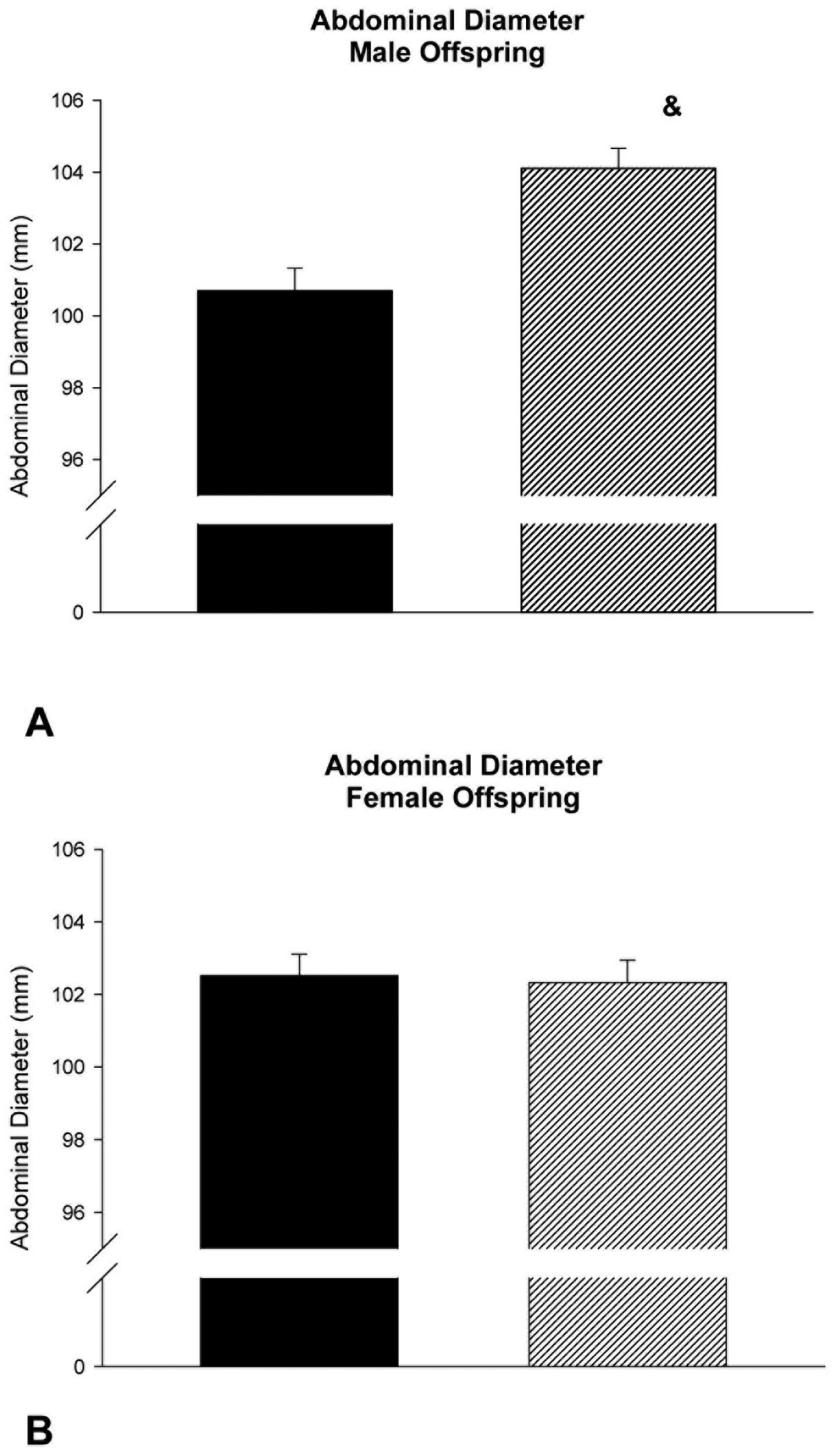
Abdominal diameter of male (A) and female (B) offspring in relationship to paternal BMI. Ultrasound examination was done in average at gestational day 266.8. Black Bar: Paternal BMI below median; Hatched Bar: Paternal BMI above median. &: p = 0.003 versus male offspring of fathers with a BMI below the median of all paternal BMI data.

However, when analyzing cases only including female offspring, all tested models revealed that paternal BMI did not correlate with birth weight, biparietal diameter, head circumference, abdominal diameter, abdominal circumference and pectoral diameter (Table 4). Key findings of the ultrasound examination are illustrated in figure 1 and 2.

**Table 4:** Multivariable regression analysis of the association between ultrasound measurements in late gestation or birth weight and paternal BMI in newborn girls.

		Model A	Model B	Model C
**Birth weight**	T	0.914	0.825	1.219
	B	6.779	6.262	8.970
	P-Value	0.362	0.410	0.224
	95%CI	−7.829–21.387	−8.689–21.213	−5.529–23.469
**Biparietal diameter**	T	1.652	1.836	1.795
	B	0.125	0.139	0.134
	P-Value	0.100	0.068	0.074
	95%CI	−0.024–0.273	−0.010–0.288	−0.013–0.280
**Head circumference**	T	1.443	1.810	1.669
	B	0.317	0.400	0.361
	P-Value	0.150	0.072	0.096
	95%CI	−0.116–0.751	−0.036–0.836	−0.065–0.787
**Abdominal diameter**	T	1.081	0.870	1.457
	B	0.170	0.139	0.228
	P-Value	0.281	0.385	0.146
	95%CI	−.140–0.479	−0.176–0.454	−0.080–0.537
**Abdominal circumference**	T	0.762	0.592	1.095
	B	0.262	0.210	0.377
	P-Value	0.447	0.554	0.275
	95%CI	−0.416–0.941	−0.489–0.908	−0.302–1.056
**Pectoral diameter**	T	−0.008	−0.015	0.245
	B	−0.001	−0.002	0.033
	P-Value	0.994	0.988	0.807
	95%CI	−0.268–0.266	−0.274–0.269	−0.230–0.295
**Femur length**	T	1.498	1.467	1.679
	B	0.097	0.097	0.107
	P-Value	0.136	0.144	0.094
	95%CI	−0.031–0.224	−0.033–0.228	−0.019–0.233

Different regression models showing the association between ultrasound measurements in late gestation or birth weight and paternal BMI in newborn girls. Shown are the T, B and P-values for paternal BMI. B: non-standardized regression coefficient.

Model A: multivariable regression analyses was done in all cases considering maternal BMI, paternal and maternal age, hypertension during pregnancy, maternal glycated serum protein, parity and eithergestational age (for birth weight) or time of ultrasound investigation (for ultrasound parameters) as confounding factors.

Model B: multivariable regression analyses was done excluding cases where fathers had hypertension but considering maternal BMI, paternal and maternal age, hypertension during pregnancy, maternal glycated serum protein, parity and either gestational age (for birth weight) or time of ultrasound investigation (for ultrasound parameters) as confounding factors.

Model C: multivariable regression analyses was done in all cases considering maternal BMI, hypertension during pregnancy, maternal glycated serum protein, and either gestational age (for birth weight) or time of ultrasound investigation (for ultrasound parameters) as confounding factors.

B: partial regression coefficient; 95% CI: 95% confidence interval for B; T: standardized regression coefficient.

These analyses uniformely showed that the effect of paternal BMI on birth weight as well on parameters describing offspring intrauterine growth are highly dependent on offspring gender.

### Effects of paternal BMI on fetal hormones

In order to detect effects of paternal BMI on the newborns endocrine system, we measured renin activity, aldosterone-, cortisol- and fetal glycated serum protein (as a parameter of glycemic control) concentrations in fetal blood taken right after delivery. Renin activity, aldosterone- and glycated serum protein concentrations of newborn boys and girls in the fetal blood right after delivery cortisol, aldosterone, renin activity and fetal glycated serum protein (as a parameter of glycemic control). Aldosterone, renin activity and glycemic control as analyzed by measuring fetal glycated serum protein were not related to paternal BMI in newborn boys and girls (data not shown). However, the cortisolCortisol concentration of newborn male offspring in newborns, however, was was significantly associated with paternal BMI in male offspring (Table 5). This association, as well as associations with birth weight and ultrasound parameters describing fetal growth were not seen in female offspring (Table 5).

**Table 5:** Multivariable regression analysis of factors predicting fetal cortisol in male and female new-born.

	Boys			Girls		
	B	95% CI	p-value	B	95% CI	p-value
**BMI Father**	11.22	0.20–22.25	0.046	6.37	−6.88–19.62	0.344
**BMI Mother**	−11.40	−24.40–1.60	0.085	−6.77	−23.07–9.53	0.413
**Age Father**	6.41	−2.08–14.90	0.138	−4.29	−13.08–4.53	0.339
**Age Mother**	−14.17	−25.71–−2.63	0.016	0.27	−12.27–12.82	0.966
**Gestational Age**	1.94	−1.52–5.38	0.272	3.88	0.51–7.19	0.024
**Hypertension during pregnancy, yes**	−67.80	−201.24–65.64	0.318	−136.94	−80.87–354.75	0.216
**Diabetes during pregnancy, yes**	8.04	−18.88–34.96	0.557	10.03	−20.45–40.50	0.517

B: partial regression coefficient; 95% CI: 95% Confidence Interval for B,

## Discussion

The present study demonstrated that paternal BMI during conception is associated with fetal growth of the male offspring but not female offspring. This was shown by analyzing birth weight and ultrasound parameter describing the body size of the newborn prior to birth. In addition, we could demonstrate that fetal cortisol at delivery is associated with paternal BMI in male offspring only.

Anthropometric parameters, especially head circumference, of newborns may be influenced by labor [26]. Measurements of anthropometric parameters were therefore obtained by ultrasound examinations some days prior to birth. Thus these values are not influenced by labor and hence are reliable. The cohort studied here is representative in terms of key characteristics of a Chinese cohort like maternal age, maternal height, maternal body mass index before pregnancy, gestational age and birth weight [27], [28] suggesting that our findings are of general impact. In order to demonstrate the robustness of our findings we used different models to analyze the relationship between paternal BMI and offspring growth. We could demonstrate that the results were similar when analyzing all cases (all comers) or just cases without any hint for an underlying paternal disease (Table 3 and 4). In addition, also the more complex model considering maternal BMI, paternal and maternal age, hypertension during pregnancy, maternal glycated serum protein concentations, parity and either gestational age (for birth weight) or time of ultrasound investigation (for ultrasound parameters) as confounding factors basically came to similar findings as the simple model considering maternal BMI, hypertension during pregnancy, maternal glycated serum protein, and either gestational age (for birth weight) or time of ultrasound investigation (for ultrasound parameters) as confounding factors (Tables 3 and 4): Paternal BMI during conception is associated with growth of the male offspring only.

The fetal programming hypothesis suggests that environmental factors in early life may alter the risk of cardiovascular diseases in later life. It is thought that environmental factors like maternal nutrition or maternal exposure to toxins like cigarette smoke lead to epigenetic alterations of the offspring which influence the activity of key cardiovascular regulatory systems like insulin resistance, blood pressure and lipid metabolism in later life [1], [29]. Typically, mechanisms leading to fetal programming of cardiovascular diseases in later life are originated from environmental influences or insults on the mother like maternal under- and over-nutrition, maternal stress, maternal exposure to toxins, and maternal genes that alter placenta function [1]. The potential roles of paternal environmental effects on offspring health, however, are poorly understood so far.

Increasing evidence emerging mainly from animal experiments indicates an important biological role of fathers in fetal programming as well. Recent studies demonstrated that high-fat diet in fathers programs β-cell dysfunction in female rat offspring via epigenetic mechanisms [20]. Also pre-mating fasting of male mice was reported to affect serum glucose levels in offspring [30]. There are so far only few human epidemiological data supporting this concept: It was shown that paternal body fat is a long-term predictor of changes in premenarch body fat of girls. There are also data linking paternal famine with a higher risk of obesity and cardiovascular disease two generations later [31], [32]. The primary aim of our study was to link paternal BMI as a surrogate for paternal obesity at conception to offspring birth weight.

Independent groups worldwide demonstrated that both maternal under-nutrition as well as maternal over-nutrition during pregnancy lead to a gender specific phenotype in the offspring [15], [33]–[38].

A recent study also indicated that paternal obesity seems to affect the offspring in a gender dependent manner [20]. Meanwhile, in various models of fetal programming it has conclusively been shown that fetal programming processes affect the offspring in a gender dependent manner. This was the justification to separately analyze female and male offspring. However, despite the knowledge that fetal programming occurs in a sex-specific manner, we just start to get insights into the underlying molecular mechanisms. The concept that environmental challenges such as nutrition challenges during pregnancy trigger stable epigenetic DNA alterations in the offspring is widely accepted providing the basis for our general understanding of molecular pathways of fetal programming. There is evidence showing that the gender dependency of fetal programming could be explained by the role of sex chromosomes, the different regulatory pathways underlying sexual development of most organs and the lifelong fluctuating impact of sex hormones. The expression of a substantial proportion of dimorphic genes is under the control of sex-specific epigenetic marks. Environmental factors such as nutrition or chemical compounds can influence – in a gender-dependent manner – these epigenetic marks during particular developmental windows of early intrauterine life. Thus, these finely tuned mechanisms may sex dependently be more or less sensitive to specific environmental challenges, particularly during developmental programming and gametogenesis [39]–[42]. These gender dependent differences in the epigenetic response to environmental factors like nutrition may cause for example gender-specific alterations of key hormone systems for cardiovascular diseases in the offspring such as the Renin-Angiotensin- Aldosterone-System (to test this hypothesis we analyzed aldosterone and renin activity in the fetal bood) or pituitary-adrenal axis (for this reason we analyzed cortisol).

To the best of our knowledge, this is the first study showing that paternal BMI during conception is associated with growth of the male but not female offspring. The underlying mechanisms still are unknown. Since we also saw a moderate association between paternal BMI and fetal cortisol in newborn boys, paternal programming of the hypothalamus-hypophyis-adrenal axis in a gender dependent manner (see above) might be possible. It was already shown that this endocrine system is subject to fetal programming [43], [44]. There is also evidence the the results seem not to be influenced by the way of taking fetal blood [45]. However, this hypothesis needs to be addressed in further studies also considering vaginal versus cesarean section and the occurrence of perinatal asphyxia besides the confounders included in our statistical model (maternal BMI, paternal and maternal age, gestational age, maternal hypertension during pregnancy and gestational diabetes, see also table 5). Our study may stimulate animal experiments aiming to explore the potential underlying mechanisms of the association of paternal BMI and male offspring intrauterine growth. It is meanwhile well established that fetal programming occurs in a gender dependent manner [15]. This was also shown in animal models of paternal programming. As mentioned above, a high-fat diet in fathers programs β-cell dysfunction in female rat offspring [20], whereas preconceptional fasting of fathers alters serum glucose particularly in male offspring of mice [30]. This study furthermore showed that in male offspring of fasted fathers, there was a possible small effect on serum corticosterone and serum IGF-1 levels. For female offspring, serum corticosterone and IGF-1 levels were not altered by preconceptional paternal fasting [30]. This also supports the hypothesis that the hypothalamus-hypophyis-adrenal axis may be subject to offspring gender specific paternal programming. Again, this needs to be proven in adequately designed experimental models.
